# Association of Rideshare Use With Alcohol-Associated Motor Vehicle Crash Trauma

**DOI:** 10.1001/jamasurg.2021.2227

**Published:** 2021-06-09

**Authors:** Christopher R. Conner, Hunter M. Ray, Ryan M. McCormack, Jacqueline S. Dickey, Samantha L. Parker, Xu Zhang, Roberto M. Vera, John A. Harvin, Ryan S. Kitagawa

**Affiliations:** 1 Department of Neurosurgery, McGovern Medical School at the University of Texas Health Science Center at Houston, Houston; 2 Department of Cardiothoracic and Vascular Surgery, McGovern Medical School at the University of Texas Health Science Center at Houston, Houston; 3 University of Texas Health Science Center at Houston School of Public Health, Houston; 4 Center for Clinical and Translational Sciences, McGovern Medical School at the University of Texas Health Science Center at Houston, Houston; 5 Department of Surgery, Baylor College of Medicine, Houston, Texas; 6 Department of Surgery, McGovern Medical School at the University of Texas Health Science Center at Houston, Houston

## Abstract

**Importance:**

Motor vehicle crashes (MVCs) are an important public health concern. Recent trends suggest that introducing rideshare services has decreased the incidence of MVCs. However, detailed analyses linking rideshare volume, convictions for impaired driving, and nonfatal MVC traumas remain inconclusive.

**Objective:**

To determine if there is an association between rideshare use and MVC traumas and convictions for impaired driving in Houston, Texas.

**Design, Setting, and Participants:**

This multicenter cohort study was conducted between January 2007 and November 2019 with hospital data from the Red Duke Trauma Institute within the Memorial Hermann Hospital–Texas Medical Center and Ben Taub General Hospital. Rideshare data from Uber and Google covered trips taken within Houston, Texas, from February 2014 (the date of deployment of Uber to Houston) to December 2018. Impaired driving convictions included all indictments made by the Harris County, Texas, District Attorney’s office from January 2007 to December 2018. All adults with MVC traumas evaluated at both centers in the study population (individuals >16 years with a mechanism of injury classified under “motor vehicle collision”) were included. Impaired driving incidents were included only if the final legal outcome was conviction.

**Main Outcomes and Measures:**

The primary study outcomes were the incident rate ratios for hourly MVC traumas and daily impaired driving convictions.

**Results:**

A total of 23 491 MVC traumas (involving patients with a mean [SD] age of 37.9 [17.8] years and 14 603 male individuals [62.1%]), 93 742 impaired driving convictions, and more than 24 million Uber rides were analyzed. Following the introduction of Uber in February 2014, MVC traumas decreased by 23.8% (from a mean [SD] of 0.26 [0.04] to 0.21 [0.06] trauma incidents per hour) during peak trauma periods (Friday and Saturday nights). The incident rate ratio of MVC traumas following Uber deployment was 0.33 (95% CI, 0.17-0.67) per 1000 indexed rides (*P* = .002). Furthermore, rideshare use was associated with a significant, geographically linked reduction in impaired driving convictions between January 2014 to December 2019 (incidence rate ratio, 0.76 [95% CI, 0.73-0.78]; *P* < .001).

**Conclusions and Relevance:**

In this study, introducing rideshare services in the Houston metropolitan area was associated with significant reductions in MVC traumas and impaired driving convictions. Increased use of rideshares may be an effective means of reducing impaired driving and decreasing rate of MVC traumas.

## Key Points

**Question:**

By decreasing impaired driving, are rideshare services associated with changes in motor vehicle trauma rates?

**Findings:**

This multicenter cohort study obtained hospital data at both major trauma centers in Houston, Texas; convictions for impaired driving from the Harris County, Texas, District Attorney’s office; and rideshare use data from Uber and Google for Houston. Rideshare volume had a significant negative correlation with the incidence of motor vehicle–associated trauma, and this was most evident in those younger than 30 years; a significant decrease in convictions for impaired driving was associated with the introduction of rideshare services.

**Meaning:**

By using rideshares to avoid impaired driving, young people may aid in decreasing motor vehicle trauma.

## Teaser

This multicenter cohort study seeks to determine if there was an association between rideshare use, motor vehicle crash traumas, and impaired driving convictions in Houston, Texas, by comparing traumas and convictions before and after the introduction of Uber.

## Introduction

Motor vehicle crashes (MVCs) are an important public health concern and a leading cause of death for people younger than 65 years.^1,2,3^ Alcohol intoxication plays a likely role in approximately one-third of MVCs and is associated with more severe injuries than unimpaired MVCs.^4,5^ Even though public policy initiatives have helped decrease alcohol-associated mortality, the prevalence of impaired driving remains high.^6^ Because MVCs have an estimated $242 billion economic cost in the US alone,^7^ all possible means of reducing the incidence of MVC traumas must be explored.

In the past decade, individuals had the choice to use a ridesharing service while socializing. Ridesharing became a promising alternative to impaired driving, in that 33% of US rideshare customers use this resource to avoid driving while impaired.^8,9^ Yet, research on the outcomes of ridesharing is limited. Previous studies have failed to capture data from nonfatal incidents.^10,11,12,13^ Unfortunately, contradictory evidence exists, in that some studies indicate that rideshares lead to an increase in MVCs,^12,13,14,15^ while others report a negative association.^4,10,16,17,18,19^ Inconclusive results in previous analyses are likely secondary to temporal granularity on the order of months, thereby lacking the detail to control daily fluctuations in rideshare use, diurnal traffic patterns, and frequency of convictions for impaired driving.^1,10,11,12,17,20^

This study aimed to quantify the change in MVC traumas and impaired driving convictions after the introduction of rideshare services. We hypothesized that increased rideshare use would correlate with decreased MVC traumas and impaired driving convictions. We evaluated specific trends and changes in hourly increments using Poisson regression by using highly granular data directly obtained from a rideshare company, Google, and hospital records. Our analysis also examined the association of rideshares with the rate of impaired driving convictions while concurrently controlling for geography, population growth, driving patterns, and alcohol consumption. This study was performed in Houston, Texas, because it is a large metropolitan area with a concentrated hospital system that has only 2 major trauma centers: Memorial Hermann Hospital–Texas Medical Center and Ben Taub General Hospital. Data from these 2 centers alone incorporate all major trauma in the Houston metropolitan area over the duration of the study.

## Methods

This work was a multicenter cohort study. This article was written in compliance with the Strengthening the Reporting of Observational Studies in Epidemiology (STROBE) guidelines.^21^ The Committee for the Protection of Human Subjects, the institutional review board at University of Texas, Houston, approved this project. Informed consent was waived because there was minimal risk to included individuals and no procedures were performed.

### Setting

Hospital data were collected in Houston at the Red Duke Trauma Institute within the Memorial Hermann Hospital–Texas Medical Center and Ben Taub General Hospital. Both are American College of Surgeons level 1 trauma centers and the only level 1 trauma centers in the greater Houston area.

### Participants, Variables, and Data Sources

Patient-level data from the MVC traumas presenting to the 2 trauma centers were retrospectively obtained from January 2007 to November 2019. Data were sourced from professionally maintained hospital trauma registries. Patients presenting with an injury mechanism classified as “motor vehicle trauma” were included regardless of outcome (fatal or nonfatal). The MVC traumas of all acuity levels were included in the analysis. Other injury mechanisms (eg, automobile-pedestrian crashes) and pediatric patients (<16 years old) were excluded. Extracted data included the time of first medical contact, demographic variables, and injury characteristics. The Injury Severity Score was used as a validated means of grading overall injury after trauma.^22^

Rideshare use data came directly from Uber (Uber Technologies Inc). These data included rides provided per hour from February 2014 (the date of deployment of Uber to Houston) to December 2018. A minimum threshold was applied and data indexed to May 6, 2014 (an index value of 1), prior to data transfer (threshold and indexing were performed by Uber, and the threshold was not disclosed to the research team). More than 24 million rides in the Houston metropolitan area were analyzed. Multiple attempts were made to obtain similar data from the rideshare company Lyft Inc without response. A second rideshare data set was obtained from Google Trends (Google LLC) search volume (http://trends.google.com), using the terms *Uber* and *Lyft*, with a temporal resolution of 1 month.^4,18,23^

A Texas Public Information Act request for impaired driving statistics was filed with the Harris County, Texas, District Attorney’s office. Arrest data for driving under the influence and driving while intoxicated included dates and locations from January 2007 to December 2019. Data were limited to incidents resulting in impaired driving guilty pleas, convictions, or probation (collectively termed *convictions* in this report), with a time resolution of days. Data from 2019 were excluded because of pending outcomes.

Notably, MVC traumas and impaired driving convictions can be biased by vehicle usage and alcohol consumption. Data concerning vehicle miles traveled in Harris County from January 2005 to December 2018 were obtained from the Texas Department of Transportation’s report on multiyear roadway data tables (https://www.txdot.gov/). Data regarding alcohol consumption for each county from January 2007 to October 2019 were gathered from the Texas Open Data Portal (http://data.texas.gov).

Total sales were inflation adjusted using the consumer price index for Harris County from the Bureau of Labor Statistics (https://www.bls.gov/). Alcohol consumption was adjusted per capita using census data (https://www.census.gov/).

### Statistical Methods

Geographic encoding was performed with GeoPy (version 1.21.0 [GeoPy Contributors], Python version 3.6.0 [Python Software Foundation]) using the Google Maps API (GoogleV3 server) and displayed in R version 3.6.2 (R Foundation for Statistical Computing)^24^ using ggmap version 3.0.0 (CRAN).^25^ Both trauma and impaired driving data were imported to R version 3.6.2 (R Foundation for Statistical Computing)^24^ as a time series. They were binned by the time of the first medical contact with a temporal resolution of 1 hour. Two outcome variables were modeled: MVC traumas and impaired driving convictions. A 0-inflated Poisson regression model was fitted for MVC traumas (using the pscl package version 1.5.5 [Political Science Computational Laboratory])^26^ because data had an hourly time resolution with many points lacking events; an uninflated Poisson regression model was fitted for impaired driving convictions at a time resolution of days (using GLM version 3.6.2 [CRAN]). The logarithm of vehicle miles traveled was used as an offset in both models. Year, month, weekday day or night, and rideshare volume (from Google Trends and Uber) were independent variables in both models. The model for impaired driving convictions also included inflation-adjusted alcohol sales per 1000 persons as an independent variable. The significance threshold was set at *P* < .05, 2-tailed. Further details can be seen in the eAppendix in the Supplement.

## Results

### Trauma Data

**Figure 1.**
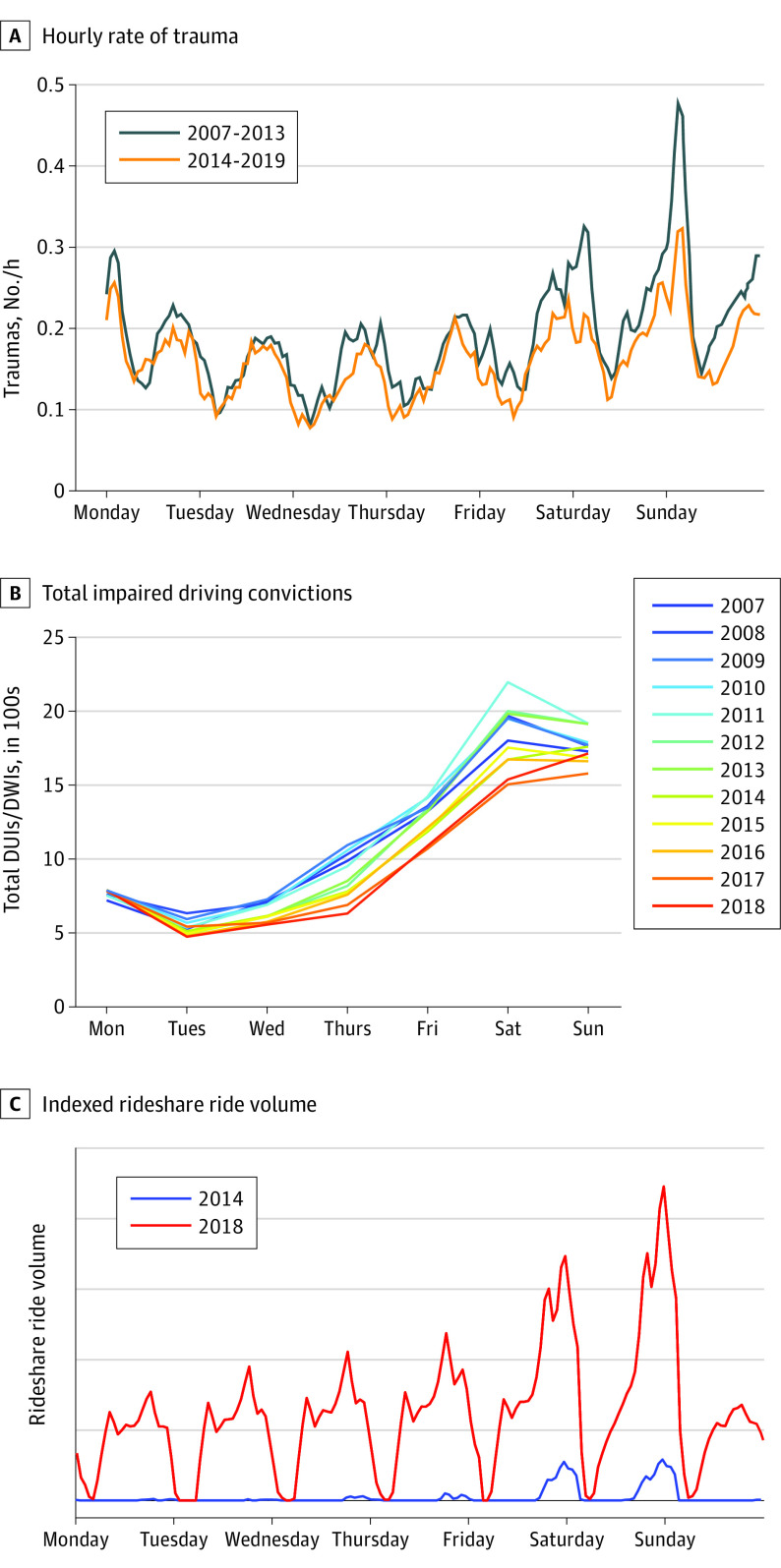
Weekly and Hourly Trends of Motor Vehicle Trauma, Impaired Driving, and Uber Usage A, Hourly rate of trauma (3-hour moving average), both before and after introducing Uber rideshare services in January 2014. B, Total impaired driving convictions (driving under the influence [DUIs] and driving while intoxicated [DWIs]) for all days in a given year, with a peak on Friday and Saturday nights. C, Indexed Uber ride volume during the first year (2014) of introduction and last year of analysis (2018). As part of the data use agreement with Uber, we were prohibited from presenting numerical ranges on the y-axis of this graph, because this was considered proprietary.

From January 2007 to November 2019, there were 23 491 MVC trauma evaluations at the 2 trauma centers (16 024 at Memorial Hermann Hospital–Texas Medical Center and 7467 at Ben Taub General Hospital). Of these, 6920 MVCs evaluations were level 1 trauma activations (the highest level of acuity) of 28 053 total level 1 traumas from all causes. Involved individuals had a mean (SD) age of 37.9 (17.8) years; 14 603 were male (62.1%). Over this period, all-cause level 1 trauma activations increased by 396 activations (20.2%), corresponding with a population increase of 1 023 997 people (26.5%) in Harris County. In 2007, there were 1911 MVC traumas, compared with 1527 from December 2018 to November 2019 (a 20.1% decrease). Regional interhospital transfer and emergency medical transport patterns to the 2 trauma centers were stable over the study period. The MVC traumas had a diurnal pattern, with the greatest numbers occurring on Friday and Saturday nights between 9 pm and 3 am. Comparison of total trauma rate from January 2007 to December 2013 (the date of Uber deployment) vs January 2014 to November 2019 revealed a 23.8% decrease in MVC traumas (from a mean [SD] of 0.26 [0.04] to 0.21 [0.06] traumas per hour) during peak hours but was otherwise unchanged (Figure 1).

**Figure 2.**
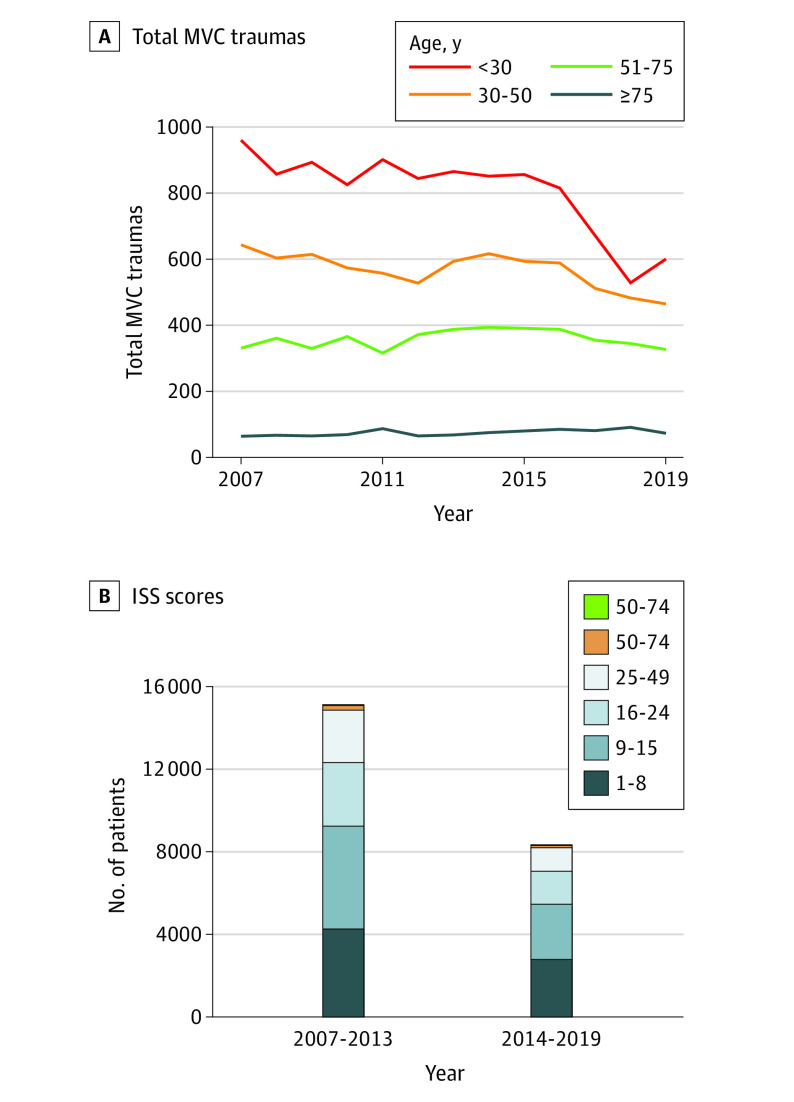
Age-Associated Changes in Motor Vehicle Crash (MVC) Incidence A, Total annual MVC traumas were calculated for 4 age ranges: younger than 30, 30-50, 51-75, and older than 75 years. Stable trauma incidence of trauma was noted across the study period, except for those younger than 30 years. Starting in 2016, there was a significant decrease in the incidence of this age range, corresponding to the introduction and increased use of rideshares post-2014 (866 in 2013 and 529 in 2018; a 38.9% reduction). B, Injury Severity Score (ISS) distributions for 2007-2013 (prerideshare) and 2014-2019 (postrideshare) also show the overall decrease in MVC incidence as well as relative decreases in higher ISS traumas. The ISS ranges are broken down into minor (1-8), moderate (9-15), serious (16-24), severe (25-49), critical (50-74), and maximal (75). Counts and percentages for ISS distributions are in eTable 4 in the Supplement.

Analysis of the MVC trauma patient demographics demonstrated significant changes in the treated patient population treated in Houston between January 2007 to December 2013 (pre–Uber introduction) and January 2014 to November 2019 (post–Uber introduction) (Table). The mean (SD) age significantly increased from 37.2 (14.4) years in the prerideshare period to 39.4 (18.3) years after Uber’s introduction (*P* < .001). The total number of patients with MVC trauma in the 4 age groups (<30, 30-50, 51-75, and >75 years) were plotted yearly (Figure 2). This demonstrated that the number of patients younger than 30 years with MVC traumas decreased after 2015 (from 866 patients in 2013 to 529 in 2018 [a 38.9% decrease]), while other age groups had stable incidence of MVC traumas.

**Table.** Population Demographics^a^

Characteristic	2007-2013	2014-2019	*P* value
Total motor vehicle crash traumas, No.	15 157	8334	NA
Age, mean (SD), y	37.2 (14.4)	39.4 (18.3)	<.001
Male, No. (%)	9475 (62.3)	5128 (61.5)	.13
Female, No. (%)	5682 (37.5)	3206 (38.5)	
Injury Severity Score, mean (SD)	15.1 (11.7)	14.0 (11.2)	.01
Survival total, No. (%)	14 582 (96.2)	8003 (96.0)	.46

Abbreviation: NA, not applicable.

^a^ Demographics for the prerideshare period (January 2007 to December 2013) and postrideshare introduction (January 2014 to November 2019) are shown, including the total number of incidents. Total motor vehicle traumas include activations of all acuity levels (level 1 and non–level 1).

The mean (SD) Injury Severity Score significantly decreased following the introduction of rideshare services (15.1 [11.7] to 14.0 [11.2]; *P* < .001). There was no difference between prerideshare and postrideshare periods in terms of distribution of sex (eg, men: prerideshare, 9475 of 15 157 [62.3%]; postrideshare, 5128 of 8334 [61.5%]; *P* = .13) or survival (14 582 [96.2%] vs 8003 [96.0%]; *P* = .46).

### Impaired Driving Data

**Figure 3.**
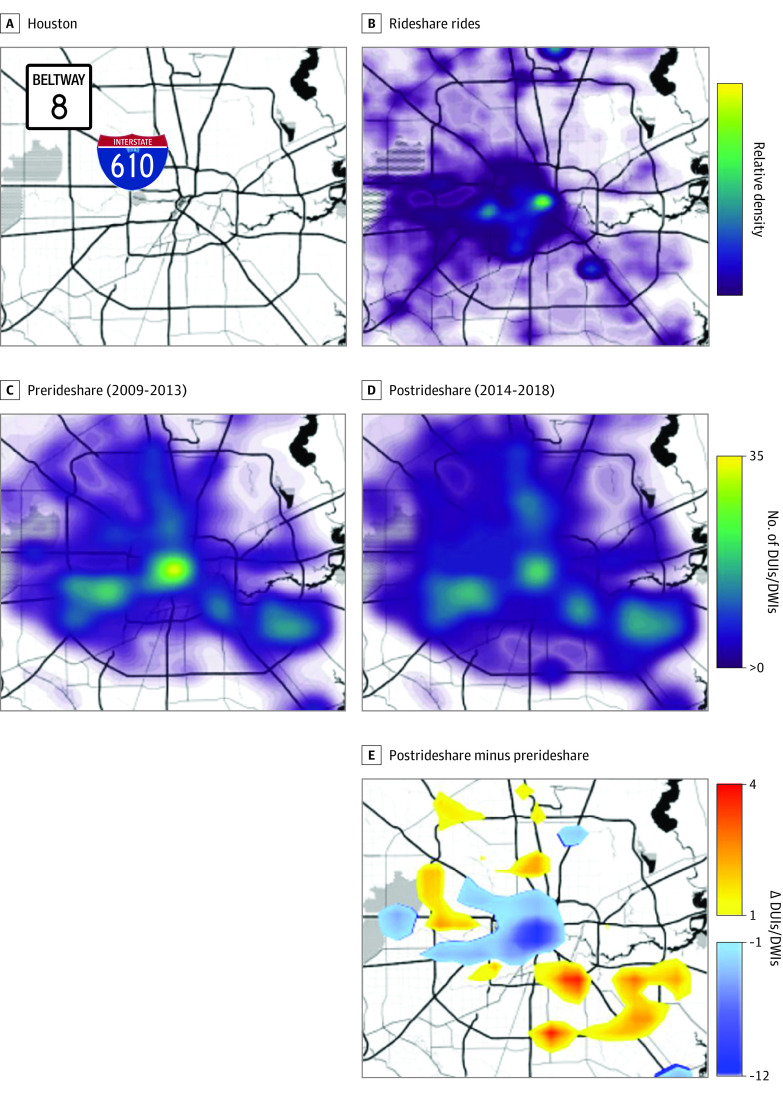
Geographic Distribution of Impaired Driving Arrests in Houston, Texas Geographic density maps were generated for Uber rides and impaired driving convictions (driving under the influence [DUIs] and driving while intoxicated [DWIs]) in Houston. A and B, Uber rides were greatest within the core of Houston (within I-610) where population is densest and around the 2 major airports. C and D, Impaired driving convictions (DUIs and DWIs) over equal-duration epochs, 2009-2013 (pre-Uber) and 2014-2018 (post-Uber), were visualized over the same map as Uber rides. Pre-Uber, convictions were highest at the city core and along major highways. Post-Uber, the peak of convictions within the core was diminished relative to convictions outside of it. E, A subtraction of post-Uber minus pre-Uber rideshares was computed geographically to better visualize the change. Decreased convictions (in blue) were noted in the core, matching with the region of greatest Uber rides. An increase (in orange) in impaired driving outside the core was seen in areas with less Uber use.

We analyzed a total of 248 485 arrests for impaired driving. Only arrests resulting in a guilty plea, conviction, or probation were included, leaving 96 520 incidents. Of these, 1294 had inaccurate location data and 1484 were duplicate records, leaving 93 742 impaired driving convictions (a category including probation and guilty pleas) for analysis. From January 2007 to December 2013, daily impaired driving convictions were unchanged, with a mean (SD) of 22.5 (10.9) per day. From January 2014 to December 2018, the rate declined to a mean (SD) of 19.0 (10.3) impaired driving convictions per day. Impaired driving convictions decreased the most for arrests occurring on Fridays, Saturdays, and Sundays (2007 vs 2018: Friday, 1323 to 1089 convictions [−17.7%]; Saturday, 1802 to 1538 convictions [−14.7%]; Sunday, 1728 to 1714 convictions [−1.0%]; Figure 1). In addition to fewer impaired driving convictions, there was a significant shift in the geographic distribution of the arrests associated with convictions. Before January 2014, impaired driving convictions stemmed from arrests predominately located in Houston’s core (encircled by the Interstate 610 loop), while post-2014 (from January 2014 to December 2018) convictions stemmed from arrests made mostly outside of Houston’s core (Figure 3).

### Seasonal Trends

**Figure 4.**
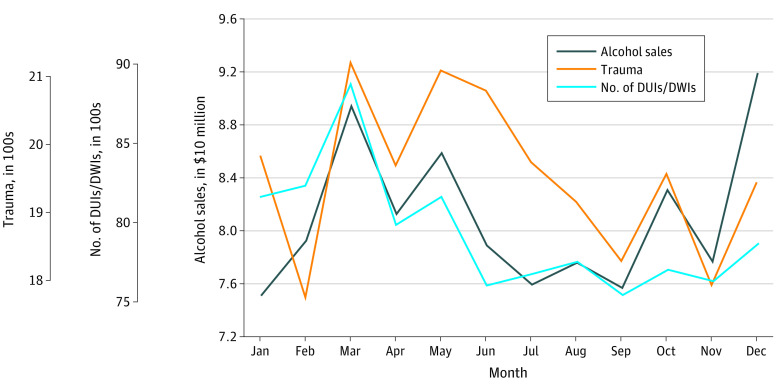
Monthly and Seasonal Trends of Motor Vehicle Crashes and Impaired Driving Black indicates Harris County, Texas, beer, wine, and liquor sales from January 2007 to October 2019, when a mean was calculated by month. March consumption is linked to the Houston Rodeo and December consumption to holidays. Blue indicates corresponding trends in impaired driving (driving under the influence [DUIs] and driving while intoxicated [DWIs]). Orange indicates motor vehicle crash traumas with a mean calculated by month.

Three months (March, May, and June) showed deviations in the number of MVC traumas. There was a corresponding increase in alcohol sales and consumption in these months (Figure 4). Seasonal variation in impaired driving convictions also increased in March and May, which corresponded with increased alcohol sales.

### Google Trends Regression Results

Poisson regression did not demonstrate a significant correlation between Google Trends searches for Uber and Lyft rideshare services and MVC traumas (eTable 1 in the Supplement). There was a significant nocturnal association with Friday and Saturday, corresponding with the greatest number of MVC traumas (rate ratios: Friday, 1.60 [95% CI, 1.49-1.72]; Saturday, 1.82 [95% CI, 1.70-1.96]; *P* < .001). This was a yearly trend after the introduction of Uber in February 2014, with significantly fewer MVC traumas after January 2016 (rate ratio, 0.63 [95% CI, 0.47-0.85]; *P* < .001).

### Uber Regression Results

Rideshare volume was associated with a 67% reduction of MVC traumas following Uber deployment (incident rate ratio, 0.33 [95% CI, 0.17-0.67] per 1000 indexed rides; *P* = .002) (eTable 2 in the Supplement). There was a significant downward trend in MVC trauma observed from January 2014 to December 2019 (rate ratio, 0.63 [95% CI, 0.57-0.68]; *P* < .001).

Moreover, there were significantly more traumas on Friday nights (rate ratio, 1.63 [95% CI, 1.57-1.69]; *P* < .001) and Saturday nights (rate ratio, 1.84 [95% CI, 1.78-1.91]; *P* < .001). Corresponding with seasonal variations, there were significantly fewer MVC trauma in September (rate ratio, 0.91 [95% CI, 0.85-0.98]; *P* = .02) and more in May (rate ratio, 1.08 [95% CI, 1.00-1.16]; *P* = .04) and June (rate ratio, 1.09 [95% CI, 1.01-1.17]; *P* = .02). Regression analysis indicated that the volume of Uber rides did not directly affect the rate of impaired driving convictions. However, we did find a significant decrease in the number of impaired driving convictions for Friday and Saturday nights (the nights with highest arrests resulting in impaired driving convictions; rate ratio, 0.72 [95% CI, 0.70-0.74]; *P* < .001; eTable 3 in the Supplement). There was also a yearly significant downward trend in impaired driving convictions following the introduction of rideshare services from February 2014 to December 2018 (rate ratio, 0.76 [95% CI, 0.71-0.80]; *P* < .001; eTable 3 in the Supplement).

## Discussion

Using 3 distinct sources of data (institutional trauma data, rideshare volume, and impaired driving convictions), this study provides the initial evidence that introducing rideshare services to the Houston area was associated with a decrease in the number of MVC traumas and impaired driving convictions. The association of rideshares with MVC traumas is 2-fold: a direct, negative association with rideshare volume and a significant yearly trend of decreasing MVCs traumas post-2014. The associations between ridesharing and impaired driving included variations by geography, driving behavior, and patterns of alcohol consumption. Previous studies have demonstrated that individuals younger than 30 years show increased Uber use rates.^27^ Our findings are consistent with those showing significant reductions in the population younger than 30 years (Table; 2013 vs 2018, 866 vs 529 [38.9%]), which traditionally is the primary age group involved in severe motor vehicle trauma (Figure 2). Since MVCs are the number 1 cause of mortality in this age group, increased use of rideshare services plays a role in preventing avoidable injuries. In older populations, Uber had no association with motor vehicle trauma, a finding that connects to the significantly lower rideshare use in these populations.

However, our study contradicts findings from some prior investigations. Previous methods include difference-in-difference comparisons,^10,12,13,14,16,18,19^ interrupted time series,^17^ and before-and-after treatment analysis.^15^ These approaches model rideshare volume as a step function. Our data indicate that these services have gained popularity in a sigmoidal pattern over 2 years. In using direct rideshare volume as a continuous regressor, there is no implicit assignment of control or treatment classes, as in difference-in-difference methods. Furthermore, this approach eliminates the temporal heterogeneity of rideshare use that limits before-after treatment analyses. Continuous data also permit a dose-dependent response estimate. As an alternative method, we used search volume for rideshare companies.^4^ We note that search volumes will aggregate rideshare trips at all times, including those not associated with impaired driving (eg, airport trips). This method also assumes that search habits are associated with ride volume.^23^ We used both methods (direct rideshare volumes and search volumes) in our analysis. We found that while significant MVC reductions occurred for peak times with direct rideshare data, search-volume data lacked statistical power. This result indicates the need for data of high temporal resolution to investigate the associations between ridesharing and MVCs.

In addition, we expected that the incidence of MVC traumas would increase during the weekends and warmer seasons (given an increase in alcohol consumption and nonsedentary activities). Indeed, both inflation-adjusted alcohol sales and MVC traumas displayed seasonal variability but with different patterns of summer increases (Figure 4). May and June are late spring and summer months in which outdoor activities increase. In Houston, an increase in MVC traumas during March may be traceable to the Houston Livestock Show and Rodeo. This event, known as *the rodeo*, annually hosts more than 2.5 million visitors.^28^

Of these, alcohol sales more closely accounted for the seasonal variability in impaired driving convictions, but the diurnal MVC traumas on a weekly time scale mirrored the known variation in impaired driving convictions (Figure 1).^29^ We conclude that the decrease in MVC traumas during both peak alcohol consumption hours and after introducing ridesharing services suggests individuals choose the ridesharing as a safe alternative to impaired driving.

Geography also played a role. Following the introduction of rideshare services, impaired driving convictions in Houston’s core saw the greatest decrease. Notably, this area also had the highest Uber service volume (Figure 3). This region’s lowered convictions still occurred, despite an increase in both the population number and density.^30^ Impaired driving convictions in outlying areas remained flat or increased, likely because of low rideshare service adoption (potentially because of higher ride costs, low driver availability, and longer wait times).

### Limitations

This study was limited by its evaluation of only a single city, resulting in potentially limited generalizability. Indeed, Houston has many unique features. First, there were only 2 level 1 trauma centers, paired with limited level 2 trauma center capacity during this period. This hospital system structure aided the study in that more traumas could be analyzed with homogenous data, but it differs from other cities with greater numbers of trauma centers and broader distribution. The lack of data from all trauma centers (level 2 and lower levels) is a limitation of the study tempered by the stability in referral patterns seen in all-cause trauma. Compared with other metropolitan areas in the US, Houston also has a lower population density, fewer public transit options, and significantly higher use of personal motor vehicles for transportation.^16,30^ It is possible that these characteristics lead to higher rates of impaired driving, in that people are more likely to imbibe further from home and with fewer options to travel. Nevertheless, the possibility that governments can reduce motor vehicle trauma by increasing accessibility to reliable, on-demand transportation should be explored further.

## Conclusions

Overall, this study indicates that introducing rideshare services to the Houston area was associated with a significant decrease in MVC trauma and impaired driving convictions. Future work will focus on the association between demographics and socioeconomic status and ridesharing services, MVC traumas, and impaired driving in other metropolitan areas.

## Notes

Supplement. **eAppendix.** Materials and Methods: Indexed Uber Rides and Statistical methods **eTable 1.** Result of ZIP regression of Google Trends ride volume estimate on MVC traumas **eTable 2.** Result of ZIP regression of hourly Uber rideshare data on MVC traumas **eTable 3.** Result of Poisson regression of daily Uber rideshare data on drunk driving convictions **eTable 4.** Injury severity score (ISS) before and after introduction of rideshares
