# Supplementary material for: Association of Rideshare Use With Alcohol-Associated Motor Vehicle Crash Trauma
Source: JAMA Surg. 2021 Jun 9;156(8):731–8. doi: 10.1001/jamasurg.2021.2227 (PMC8190695; doi:10.1001/jamasurg.2021.2227)
Supplement: Supplement. — eAppendix. Materials and Methods: Indexed Uber Rides and Statistical methods eTable 1. Result of ZIP regression of Google Trends ride volume estimate on MVC traumas eTable 2. Result of ZIP regression of hourly Uber rideshare data on MVC traumas eTable 3. Result of Poisson regression of daily Uber rideshare data on drunk driving convictions eTable 4. Injury severity score (ISS) before and after introduction of rideshares [file jamasurg-e212227-s001.pdf]

Supplemental Online Content

Conner CR, Ray HM, McCormack RM, et al. Association of rideshare use with alcohol-associated motor vehicle crash trauma. *JAMA Surg*. Published online June 9, 2021. doi:10.1001/jamasurg.2021.2227

eAppendix. Materials and Methods: Indexed Uber Rides and Statistical methods
eTable 1. Result of ZIP regression of Google Trends ride volume estimate on MVC traumas
eTable 2. Result of ZIP regression of hourly Uber rideshare data on MVC traumas
eTable 3. Result of Poisson regression of daily Uber rideshare data on drunk driving convictions
eTable 4. Injury severity score (ISS) before and after introduction of rideshares

This supplemental material has been provided by the authors to give readers additional information about their work.

© 2021 American Medical Association. All rights reserved.

eAppendix.
Materials and Methods

Index Uber Rides
Hourly rideshare volume data were supplied directly from Uber. Data were supplied from introduction of the service to Houston in February 2014 through the end of 2018. Hourly rides were thresholded above a minimum ride volume and then indexed relative to May 6, 2014 (index value of 100).

Statistical Methods
Data were imported into R (ver 3.6.2, CRAN and R Foundation for Statistical Computing) as time series and binned by time of first medical contact with a temporal resolution on the order of 1 hour. Two outcome variables were modeled: MVC traumas and drunk driving convictions (DUIs/DWIs). The zero-inflated Poisson (ZIP) regression model was fitted for MVC traumas and the Poisson regression model with a dispersion parameter was fitted for DUIs/DWIs. Logarithm of VMT was used as an offset in both models. Year, month, weekday day/night, and rideshare volume (from Google Trends and Uber) were the regressors in both models. The model for DUIs/DWIs also included inflation-adjusted alcohol sales per 1,000 persons as a regressor. All analyses were performed in R.

© 2021 American Medical Association. All rights reserved.

eTable 1. Result of ZIP regression of Google Trends ride volume estimate on MVC traumas.

Results are reported as regression coefficient and standard error estimate from zero-inflated Poisson model. To estimate the rate ratio, the natural exponent of the regression coefficient is calculated.

Variable	Regression coefficient estimate (standard error)	p value
Google trends (Uber + Lyft)	$-7.1x10^{-4}$ ($1.4x10^{-3}$)	p=0.61
Year effect (reference year = 2007)		
2008	$2.1x10^{-2}$ ($3.5x10^{-2}$)	p=0.54
2009	$3.1x10^{-2}$ ($3.5x10^{-2}$)	p=0.37
2010	$-4.0x10^{-2}$ ($3.5x10^{-2}$)	p=0.26
2011	$-6.3x10^{-2}$ ($3.6x10^{-2}$)	p=0.08
2012	$-9.0x10^{-2}$ ($3.6x10^{-2}$)	p=0.01
2013	$-3.7x10^{-2}$ ($3.6x10^{-2}$)	p=0.30
2014	$-3.6x10^{-2}$ ($4.7x10^{-2}$)	p=0.45
2015	$-1.4x10^{-1}$ ($8.9x10^{-2}$)	p=0.12
2016	$-2.6x10^{-1}$ ($1.2x10^{-1}$)	p=0.04
2017	$-3.7x10^{-1}$ ($1.4x10^{-1}$)	p<0.01
2018	$-4.6x10^{-3}$ ($1.5x10^{-1}$)	p<0.002
Month effect (reference month = January)		
February	$4.8x10^{-3}$ ($3.8x10^{-2}$)	p=0.90
March	$6.6x10^{-2}$ ($3.7x10^{-2}$)	p=0.07
April	$2.7x10^{-2}$ ($3.7x10^{-2}$)	p=0.46
May	$7.8x10^{-2}$ ($3.7x10^{-2}$)	p=0.03
June	$9.0x10^{-2}$ ($3.8x10^{-2}$)	p=0.02
July	$-7.6x10^{-2}$ ($3.8x10^{-2}$)	p=0.84
August	$-5.0x10^{-2}$ ($3.9x10^{-2}$)	p=0.20
September	$-9.0x10^{-2}$ ($3.9x10^{-2}$)	p=0.02
October	$-2.0x10^{-2}$ ($3.8x10^{-2}$)	p=0.60
November	$-6.0x10^{-3}$ ($3.8x10^{-2}$)	p=0.88
December	$4.2x10^{-2}$ ($3.8x10^{-2}$)	p=0.22
Weekday effect (reference = Sunday day)		
Sunday night	$-1.0x10^{-3}$ ($4.0x10^{-2}$)	p=0.80
Monday day	$-6.6x10^{-2}$ ($4.1x10^{-2}$)	p=0.10
Monday night	$-3.7x10^{-1}$ ($4.4x10^{-2}$)	p<0.01
Tuesday day	$-9.0x10^{-2}$ ($4.1x10^{-2}$)	p=0.03
Tuesday night	$-3.0x10^{-1}$ ($4.4x10^{-2}$)	p<0.01
Wednesday day	$-1.4x10^{-1}$ ($4.2x10^{-2}$)	p<0.01
Wednesday night	$-2.3x10^{-1}$ ($4.3x10^{-2}$)	p<0.01
Thursday day	$-7.8x10^{-2}$ ($4.1x10^{-2}$)	p=0.06
Thursday night	$-3.7x10^{-2}$ ($4.1x10^{-2}$)	p=0.36
Friday day	$1.0x10^{-2}$ ($4.0x10^{-2}$)	p=0.79
Friday night	$4.7x10^{-1}$ ($3.7x10^{-2}$)	p<0.01
Saturday day	$3.1x10^{-2}$ ($4.0x10^{-2}$)	p=0.44
Saturday night	$6.0x10^{-1}$ ($3.6x10^{-2}$)	p<0.01

© 2021 American Medical Association. All rights reserved.

eTable 2. Result of ZIP regression of hourly Uber rideshare data on MVC traumas.

Results are reported as regression coefficient and standard error estimates from zero-inflated Poisson model.

Variable	Regression coefficient estimate (standard error)	p value
Rideshare volume	-1.1×10^{-3} (3.5×10^{-4})	p=0.002
Year effect (reference year = 2007)		
2008	2.1×10^{-2} (3.5×10^{-2})	p=0.54
2009	3.2×10^{-2} (3.5×10^{-2})	p=0.46
2010	-4.0×10^{-2} (3.6×10^{-2})	p=0.26
2011	-6.3×10^{-2} (3.6×10^{-2})	p=0.08
2012	-9.0×10^{-2} (3.6×10^{-2})	p=0.01
2013	-3.8×10^{-2} (3.6×10^{-2})	p=0.28
2014	-4.9×10^{-2} (3.6×10^{-2})	p=0.17
2015	-1.6×10^{-1} (3.7×10^{-2})	p<0.01
2016	-2.8×10^{-1} (3.9×10^{-2})	p<0.01
2017	-3.8×10^{-1} (4.3×10^{-2})	p<0.01
2018	-4.7×10^{-3} (4.3×10^{-2})	p<0.01
Month effect (reference month = January)		
February	3.9×10^{-2} (3.7×10^{-2})	p=0.92
March	6.5×10^{-2} (3.6×10^{-2})	p=0.08
April	2.6×10^{-2} (3.7×10^{-2})	p=0.48
May	7.5×10^{-2} (3.6×10^{-2})	p=0.04
June	8.5×10^{-2} (3.7×10^{-2})	p=0.02
July	-1.2×10^{-2} (3.7×10^{-2})	p=0.75
August	-5.5×10^{-2} (3.8×10^{-2})	p=0.14
September	-9.1×10^{-2} (3.7×10^{-2})	p=0.02
October	-2.0×10^{-2} (3.7×10^{-2})	p=0.58
November	-8.6×10^{-2} (3.7×10^{-2})	p=0.81
December	4.2×10^{-2} (3.7×10^{-2})	p=0.25
Weekday effect (reference = Sunday day)		
Sunday night	-4.4×10^{-3} (4.0×10^{-2})	p=0.91
Monday day	-6.6×10^{-2} (4.1×10^{-2})	p=0.11
Monday night	-3.6×10^{-1} (4.4×10^{-2})	p<0.01
Tuesday day	-8.9×10^{-2} (4.1×10^{-2})	p=0.03
Tuesday night	-2.9×10^{-1} (4.4×10^{-2})	p<0.01
Wednesday day	-1.4×10^{-1} (4.2×10^{-2})	p<0.01
Wednesday night	-2.2×10^{-1} (4.3×10^{-2})	p<0.01
Thursday day	-7.7×10^{-2} (4.1×10^{-2})	p=0.06
Thursday night	-2.2×10^{-2} (4.1×10^{-2})	p=0.60
Friday day	1.4×10^{-2} (4.0×10^{-2})	p=0.72
Friday night	4.9×10^{-1} (3.7×10^{-2})	p<0.01
Saturday day	3.5×10^{-2} (4.0×10^{-2})	p=0.38
Saturday night	6.1×10^{-1} (3.6×10^{-2})	p<0.01

© 2021 American Medical Association. All rights reserved.

eTable 3. Result of Poisson regression of daily Uber rideshare data on drunk driving convictions.

Results are reported as regression coefficient and standard error estimates from Poisson regression model with a dispersion parameter. Alcohol sales are $1 per person-year and inflation adjusted.

Variable	Regression coefficient estimate (standard error)	p value
Rideshare volume	-1.5×10^{-2} (1.5×10^{-2})	p=0.29
Alcohol Sales	2.6×10^{-2} (5.9×10^{-3})	p<0.01
Year effect (reference year = 2007)		
2008	1.1×10^{-1} (1.9×10^{-2})	p<0.01
2009	1.8×10^{-1} (2.1×10^{-2})	p<0.01
2010	1.2×10^{-1} (2.0×10^{-2})	p<0.01
2011	1.4×10^{-1} (1.9×10^{-2})	p<0.01
2012	7.1×10^{-2} (1.9×10^{-2})	p<0.01
2013	6.0×10^{-2} (2.0×10^{-2})	p=0.002
2014	-8.0×10^{-4} (2.1×10^{-2})	p=0.97
2015	-9.4×10^{-2} (2.1×10^{-2})	p<0.01
2016	-2.1×10^{-1} (2.5×10^{-2})	p<0.01
2017	-2.5×10^{-1} (2.8×10^{-2})	p<0.01
2018	-2.8×10^{-1} (2.8×10^{-2})	p<0.01
Month effect (reference month = January)		
February	9.0×10^{-2} (2.0×10^{-2})	p<0.01
March	2.0×10^{-2} (2.6×10^{-2})	p=0.45
April	6.9×10^{-3} (2.1×10^{-2})	p=0.74
May	-2.7×10^{-2} (2.4×10^{-2})	p=0.26
June	-1.9×10^{-2} (2.0×10^{-2})	p=0.35
July	-2.1×10^{-2} (2.0×10^{-2})	p=0.30
August	-6.8×10^{-3} (2.0×10^{-2})	p=0.73
September	2.0×10^{-2} (2.0×10^{-2})	p=0.31
October	-2.9×10^{-2} (2.2×10^{-2})	p=0.19
November	9.1×10^{-3} (2.0×10^{-2})	p=0.65
December	-7.3×10^{-2} (3.1×10^{-2})	p=0.02
Weekday effect (reference day = Sunday)		
Monday	-8.2×10^{-1} (1.5×10^{-2})	p<0.01
Tuesday	-1.2 (1.7×10^{-2})	p<0.01
Wednesday	-1.0 (1.6×10^{-2})	p<0.01
Thursday	-7.1×10^{-1} (1.4×10^{-2})	p<0.01
Friday	-3.3×10^{-1} (1.3×10^{-2})	p<0.01
Saturday	3.9×10^{-2} (1.1×10^{-2})	p=0.001

© 2021 American Medical Association. All rights reserved.

eTable 4. Injury severity score (ISS) before and after introduction of rideshares.

ISS for the pre-rideshare period (2007-2013) and post-rideshare introduction (2014-2019) are shown in total number of patients.

ISS (percent)	2007-2013	2014-2019
1-8 (minor)	4253 (28.1)	2781 (33.4)
9-15 (moderate)	4984 (33.0)	2676 (32.1)
16-24 (serious)	3084 (20.4)	1606 (19.3)
25-49 (severe)	2537 (16.8)	1133 (13.6)
50-74 (critical)	212 (1.4)	101 (1.2)
75 (maximum)	56 (0.4)	37 (0.4)

© 2021 American Medical Association. All rights reserved.
